# Honey induces apoptosis in renal cell carcinoma

**DOI:** 10.4103/0973-1296.75901

**Published:** 2011

**Authors:** Saeed Samarghandian, Jalil Tavakkol Afshari, Saiedeh Davoodi

**Affiliations:** *Department of Physiology, Mashhad University Medical Sciences, Mashhad,, Iran*; 1 *Immunology Research Center, BuAli Research Institute, Mashhad University of Medical Sciences, Mashhad, Iran*

**Keywords:** ACHN, Annexin-V, Apoptosis, honey, MTT

## Abstract

**Background:**

The fact that antioxidants have several preventative effects against different diseases, such as coronary diseases, inflammatory disorders, neurologic degeneration, aging, and cancer, has led to the search for food rich in antioxidants. Honey has been used as a traditional food and medical source since ancient times. However, recently many scientists have been concentrating on the antioxidant property of honey. By use of human renal cancer cell lines (ACHN), we investigated the antiproliferative activity, apoptosis, and the antitumor activity of honey.

**Materials and Methods:**

The cells were cultured in Dulbecco’s modified Eagle’s medium with 10% fetal bovine serum treated with different concentrations of honey for 3 consecutive days. Cell viability was quantitated by the 3-(4,5-Dimethylthiazol-2-yl)-2,5-diphenyltetrazolium bromide assay. Apoptotic cells were determined using Annexin-V-fluorescein isothiocyanate (FITC) by flow cytometry.

**Results:**

Honey decreased the cell viability in the malignant cells in a concentration- and time-dependent manner. The IC _50_ values against the ACHN cell lines were determined as 1.7 ± 0.04% and 2.1 ± 0.03% μg/mL after 48 and 72 h, respectively. Honey induced apoptosis of the ACHN cells in a concentration-dependent manner, as determined by flow cytometry histogram of treated cells.

**Conclusion:**

It might be concluded that honey may cause cell death in the ACHN cells, in which apoptosis plays an important role. Most of the drugs used in the cancer treatment are apoptotic inducers, hence apoptotic nature of honey is considered vital. Therefore, it prompted us to investigate honey as a potential candidate for renal cancer treatment.

## INTRODUCTION

Renal cancer accounts for nearly 2% of all malignancies globally. The American Cancer Society estimated approximately 36,160 new cases of kidney cancer in 2005. More than 80% of kidney cancers are renal cell carcinomas (RCC), the remainder being mainly renal pelvis cancers. Mortality to incidence ratio of RCC is higher compared with other urologic malignancies. RCC is erratic and unpredictable even when diagnosed and treated early by nephrectomy, as the neoplasm can appear to remain stable for years and then metastasize to distant locations[1] Renal cell carcinoma is characterized by a lack of specific clinical signs that allow the diagnosis at an early stage.[2] Therefore, a high proportion of patients will have metastasis at the time of first diagnosis and cannot be cured, because there is no effective therapy for metastatic renal cancer. Traditional therapeutic approaches to RCC, such as radiotherapy, chemotherapy, or hormone- therapy, have little or no effect on this disease,[2] although immune-modulating agents, cytokines, or differentiating agents, such as retinoids, have shown antitumor activity in a small proportion of patients with metastatic renal cancer.[3–5] These methods do not address metastases and can promote tumor progression by impairing the immune system. Treatment methods focused on the regulation of tumor proliferation are necessary to control RCC effectively.

The treatment of RCC has made little progress in the past 30 years and no chemotherapeutic agents currently available are effective against it.[6,7] There is a need for novel and more selective drugs that are able to interfere with targets directly involved in the process of renal cancer development and progression. On the other hand, the biological heterogeneity of RCC, its resistance to anti-cancer drugs, and the side effects of chemotherapeutics are the major obstacles in the effective treatment of RCC. Radical nephrectomy of localized RCC is effective only in a few cases because the rate of systemic metastasis is high with nearly 50% of the patients developing metastasis after surgical resection.[8,9] Patients with metastatic RCC have a median survival rate of 10 months and <2% of patients survive beyond 5 years.[8] Therefore, the search for effective therapeutic agents for this malignancy is urgently needed.

Antioxidant-rich foods have several preventive effects against different diseases, such as cancer, coronary disease, inflammatory disorders, and neurologic degeneration.[10,11] Honey has been used as a traditional food source since ancient times. Honey is the substance made when the nectar and sweet deposits from plants are gathered, modified, and stored in the honeycomb by honeybees. The major components of honey are fructose and glucose and also it consists of carbohydrates, proteins, amino acids, vitamins, water, minerals, and enzymes. In general, honey is also rich in antioxidants[12,13] and has antibacterial properties.[14,15] There are many reports in the medical literature of honey being effective as a dressing for wounds,[16–21] burns,[22–25] and ulcer.[26,27,28,29] Honey not only promotes growth of new skin tissue by creating a moist environment, but also prevents infection by way of its antimicrobial properties. Moreover, honey is harmless and in fact enables faster healing of the wounds by forming new tissues.

Honey is thought to exhibit a broad spectrum of therapeutic properties, including antibacterial, antifungal, cytostatic, and anti-inflammatory activity.[30] Honey has been used for the treatment of Fournier’s gangrene, abdominal wound disruption, gastric ulcers,[31] gastroenteritis, and burns and for the storage of skin grafts.[32] Recent studies by Gribel and Pashiniski indicated that honey possessed moderate antitumor and pronounced antimetastatic effects in 5 different strains of rat and mouse tumors.[33] Furthermore, honey potentiated the antitumor activity of chemotherapeutic drugs, such as 5-fluorouracil and cyclophosphamide.[34] Honey contains many biologically active compounds, including caffeic acid, caffeic acid phenethyl ester, and flavonoid glycones. These compounds have been proved to have an inhibitory effect on tumor cell proliferation and transformation by the downregulation of many cellular enzymatic pathways, including protein tyrosine kinase, cyclooxygenase, and ornithine decarboxylase pathways.[35]

Recently, Tarek *et al*. showed that honey could induce apoptosis inT24, RT4, 253J, and MBT-2 bladder cancer cell lines. They showed significant inhibition of the proliferation of T24 and MBT-2 cell lines by 1%—25% honey and of RT4 and 253J cell lines by 6%—25% honey. Further in the *in vivo* studies, intralesional injection of 6% and 12% honey, as well as oral ingestion of honey significantly inhibited tumor growth.[36] Developments of new drugs with better efficacy are gaining momentum. The search for food as medicine is constantly evolving and people exploit various antioxidant-rich foods for this purpose. Therefore, the present study provides an updated overview of experimental *in vitro* investigation on the biological activities of honey, especially focusing on its cytotoxicity toward the ACHN renal cancer cell line.

## MATERIALS AND METHODS

### Chemicals and reagents

3-(4,5-Dimethylthiazol-2-yl)-2,5-diphenyltetrazolium bromide (MTT) was purchased from Amerco (Nevada, USA). RPMI 1640 was purchased from Gibco BRL (Grand Island, NY, USA). Annexin-V-FITC was obtained from Invitrogen Corporation (Camarillo, CA, USA). Fetal bovine serum was purchased from PAA Laboratories GmbH, Pasching, Austria. The crude brand of honey was stored at 4°C and its manufacturing date was under 2 months while performing the experiments. The sample originated from Khorasan region of Iran. According to the manufacturers’ details, all the honey types were considered multi-floral.

### Cell culture

The renal carcinoma cell lines, ACHN, were obtained from the Pasteur Institute, Tehran, Iran. The cells were grown either in 96-well tissue (TC) plate (NUNC, Wiesbaden, Germany) or in 25 cm^2^ flasks (NUNC, Wiesbaden, Germany), cultured in RPMI medium supplemented with 10% FBS (Gibco-Invitrogen), 100 U/ml of penicillin (Gibco-Invitrogen), and 100 μg/mL streptomycin (Gibco-Invitrogen). ACHN cells were cultured in CO_2_ incubator MCO-17AI (Sanyo Electric Co., Ltd, Japan) at 37°C; in 95% humidified atmosphere enriched by 5% CO_2_ and subcultured every 3–4 days.

### Cell viability assay

Cell viability was measured using the MTT assay, which is based on the conversion of MTT to formazan crystals by mitochondrial dehydrogenases.[37] Briefly, ACHN cells were plated at a density of (1 ×10^3^ cells/mL) in 96-well plates and allowed to attach for 24 h to keep the log phase growth at the time of drug treatment. Honey at different concentrations (2.5%, 5%, 10%, 20%) was added to the wells for 24, 48, and 72 h. After treatment with honey for 72 h, 10 μL MTT was added into each well. After 4 h incubation at 37°C, this solution was removed, and the produced formazan was solubilized in 100 μL dimethyl sulfoxide. Absorbance was measured at 550 nm using an automated microplate reader (Bio-Rad 550, California, USA). Cell viability was expressed as a percentage of the control culture value. The cytotoxic effects of honey on ACHN cell line was expressed as IC _50_ value (the drug concentration reducing the absorbance of treated cells by 50% with respect to untreated cells). All experiments were carried out in triplicate.

### Morphologic studies of cell lines using the normal inverted microscope

Morphologic studies using the normal inverted microscope were carried out to observe the morphologic changes of cell death in ACHN cell lines elicited by honey. Different concentrations of (2.5%, 5%, 10%, 20%) of honey for 24, 48, and 72 h were used for the morphologic studies. The untreated cells served as the negative control. The morphologic changes of the cells were observed under the normal inverted microscope after 24 and 48 h post-treatment.

### Assessment of apoptosis by Annexin-V-FITC

Apoptotic cell death caused by honey was measured using FITC-conjugated Annexin-V/PI assay kit by flow cytometry,[38] briefly 5 × 10^5^ cells were washed with icecold phosphate buffer solution, resuspended in 100 μL binding buffer, and stained with 5 μL of FITC-conjugated Annexin-V (10 mg/mL) and 10 μL of PI (50 mg/mL). The cells were incubated for 15 min at room temperature in the dark, and then 400 μL of binding buffer was added, and analyzed by a FACScan flow cytometry (Becton--Dickinson, Franklin Lakes, NJ USA). For analysis, ACHN cell lines were gated separately according to their granularity and size on forward scatter vs side scatter plot. Early apoptosis and late apoptosis were evaluated on fluorescence 2 (FL2 for propidium iodide) vs fluorescence 1 (FL1 for Annexin) plots. The percentage of cells stained with Annexin-V only was evaluated as early apoptosis; the percentage of cells stained with both Annexin-V and propidium iodide was evaluated as late apoptosis or necrotic stage.

### Statistical analysis

All results were expressed as mean ± SEM. The significance of difference was evaluated with ANOVA and Bonferroni’s test.[39] A probability level of *P* < 0.05 was considered statistically significant.

## RESULTS

### Effects of honey on cell viability

**Figure 1:**
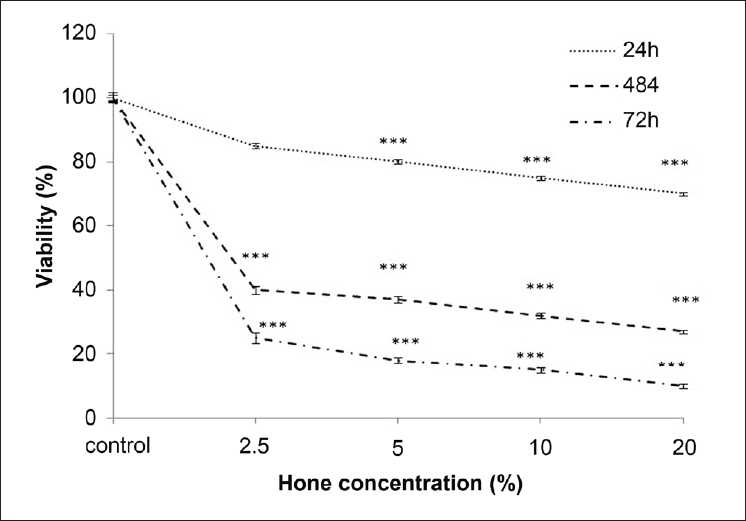
Effect of honey extract on cell viability of ACHN cells. Cells were treated with different concentrations of honey extract for 24, 48, and 72 h. Viability was quantitated by MTT assay. Results are mean ± SEM. The asterisks (***P < 0.001) are indicator of statistical difference obtained separately at different time points compared with their controls

ACHN cell line was incubated with various concentrations of honey for 24, 48, and 72 h. The impact of honey on the cell viability was quantitated by MTT assay. Exposure of the ACHN cells for 24, 48, and 72 h with honey showed significantly high growth inhibitory effects on renal carcinoma cell line in a concentration- and time-dependent manner (*P* < 0.001). Although there was no significant result at a low concentration of honey (2.5%) after 24 h, the exposure of ACHN cell line for 24 h decreased the number of viable cells at higher doses of honey (5%, 10%, 20%) (*P* < 0.001) vs control. On the other hand, treatment of ACHN cell lines for 48 and 72 h with different doses of honey (2.5%, 5%, 10%, 20%) resulted in a marked reduction in the number of viable cells (*P* < 0.001) [Figure 1]. The dose inducing 50% cell growth inhibition (IC_50_) against malignant cells was determined at 1.7% ± 0.04% and 2.1% ± 0.03% after 48 and 72 h, respectively [Table 1].

**Table 1:** Doses inducing 50% cell growth inhibition (IC_50_) of honey against the renal carcinoma cell lines (ACHN)

IC_50_	48 h	72 h
ACHN	1.7±0.04%	2.1±0.03%

Cells were treated with different concentrations of honey for 24, 48, and 72 h. Viability was quantitated by MTT assay

### Morphologic evaluation

**Figure 2:**
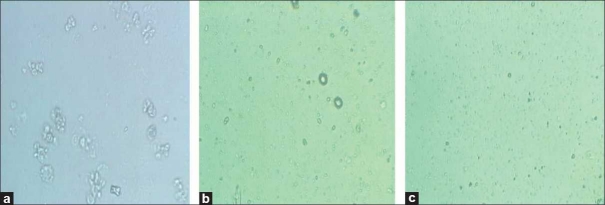
Comparison of cytotoxicity effect of honey extract on cell viability of ACHN. Morphologic changes of cells after treatment with different concentrations of honey after 48 h. [a = normal cells; b = treatment with honey (2.5%); c = treatment with honey (20%)]

Although the morphologic features were not significantly changed after 24 h of incubation with low doses of honey (2.5%) (data was not shown), after 48 h of incubation with concentrations of 2.5% and 20% of honey, morphologic changes were observed using the normal inverted microscope compared with those of the control. Cells treated with honey (20%) showed a more prominent growth inhibition and shrinkage of the cells compared with the cells treated with a lower dose of honey (2.5%), which consisted of induction in a number of living cells, volume and rounding [Figure 2] and confirmed our MTT results.

### Quantification studies for apoptosis by honey

**Figure 3:**
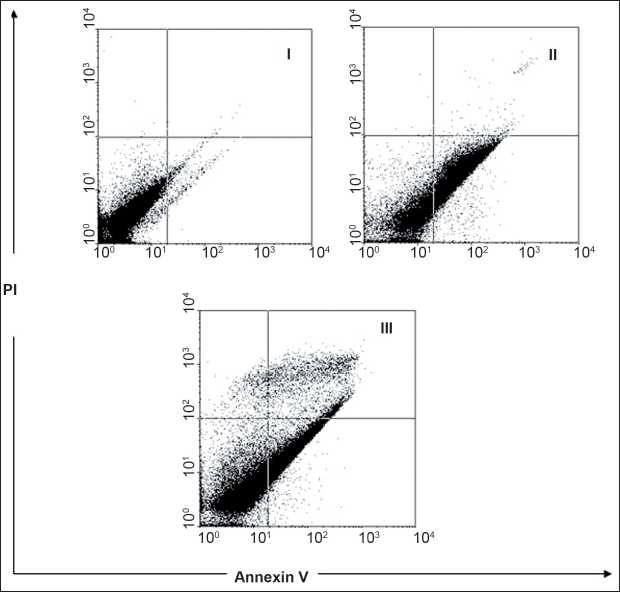
Assessment of apoptosis by Annexin-V/PI on the renal cell carcinoma (ACHN). The cells were treated with different concentrations of honey (2.5% and 20%) for 48 h (II and III, respectively) or media (control symbol I), and apoptosis was examined with flow- cytometry after Annexin-V/PI double staining. The necrotic cells lost cell membrane integrity that permits PI entry. Viable cells exhibit Annexin-V/PI; early apoptotic cells exhibit Annexin-V/PI −; and late apoptotic cells or necrotic cells exhibit Annexin-V+/PI+

**Figure 4:**
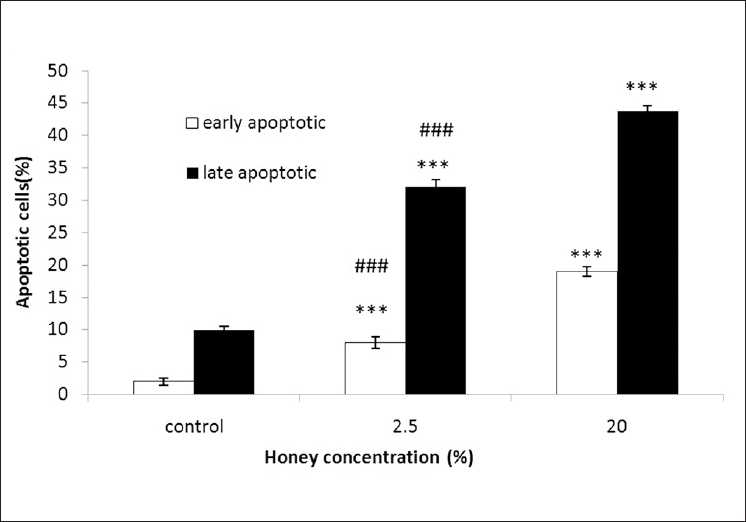
Assessment of apoptosis by Annexin-V/PI on renal cell carcinoma (ACHN). Percentage of cell death based on the assessment of apoptosis by Annexin-V/PI. ***and ###*P* < 0.001 compared with the control and the other dose, respectively

To study the role of honey in apoptosis, honey was used to set up apoptosis system on ACHN cell lines. ACHN cells were treated with concentrations of 2.5% and 20% honey for 48 h. After treatment, the cells were harvested and apoptosis was examined by flow cytometry [Figure 3]. Quantitative analysis using Annexin-V/PI assay further showed that the proportion of the early-stage apoptotic cells (Annexin-V+/PI–-) increased significantly from 2.0% to 19.0%, while the proportion of the late-stage apoptotic cell (Annexin-V+/PI+) increased significantly from 10.0% to 43.8% [Figure 4]. Apoptosis induced from 2.5% and 20% of honey was statistically higher than that in the control, and the percentage of the early and late apoptotic cells significantly increased by increasing honey concentration (*P* < 0.001). The number of late apoptotic cells vs early apoptotic cells at concentrations of 2.5% and 20% honey-treated cells were statistically significant (P < 0.001), so that, the percentage of the late apoptotic cells increased significantly compared with the percentage of the early apoptotic cells [Figure 4].

## DISCUSSION

Epidemiologic surveys and experimental studies have provided evidence that environmental factors, including dietary substances, play a major role in the incidence of cancer. Natural products are perceived as pure, and without side effects as medication products.[38] Many patients with cancer or other chronic conditions use alternative therapies, often herbal or natural products.[39–44] Honey, which is one of the most complex mixtures of carbohydrates produced in nature,[45] has a long history as a medicinal substance.[46] It is a known natural product with several biological activities.[47,48]

Honey has been used since a long time both for medical and domestic needs, but only recently the antioxidant property of it came to limelight. The fact that antioxidants have several preventative effects against different diseases, such as cancer, coronary diseases, inflammatory disorders, neurologic degeneration, and aging, has led to a search for foods rich in antioxidants. Chemoprevention uses various dietary agents rich in phytochemicals, which serve as antioxidants. With increasing demand for antioxidant supply in the food, honey has gained prominence because it is rich in phenolic compounds and other antioxidants, such as ascorbic acid, amino acids, and proteins. Some simple and polyphenols found in honey, namely, caffeic acid (CA), caffeic acid phenyl esters (CAPE), Chrysin (CR), Galangin (GA), Quercetin (QU), Kaempferol (KP), Acacetin (AC), Pinocembrin (PC), Pinobanksin (PB), and Apigenin (AP), have evolved as promising pharmacologic agents in the treatment of cancer. Some bioactive compounds, such as Chrysin, have been found in honey, which have been used to prevent cancer, in a similar fashion as anastrozole (a breast cancer drug), and treat conditions, such as anxiety and inflammation.[49–50] Honey is also known as a dietary source for flavonoids,[51] which have been demonstrated to have anti-carcinogenic and anti-inflammatory activities.[52] Although crude honey was reported by some authors as a proliferative agent that enhances the proliferation of both normal and malignant cells,[53–55] it was also reported as a promising antitumor agent with pronounced antimetastatic effect.[56,57] The proliferative effect of honey on tumor cells was suggested to be a nutritional effect rather than a carcinogenic effect and the antitumor effect was reported to result from many activities, such as the inhibition of DNA synthesis with no signs of cytotoxicity and downregulation of MMP-2 and MMp-9, which have been implicated in the induction of the angiogenic switch in different model systems.[58]

In this study, the cytotoxic and proapoptotic effects of honey in ACHN cell lines were investigated. To the authors’ knowledge, this is the first report on honey-induced apoptosis in human renal adenocarcinoma cell line. Our data confirmed that honey has cytotoxic activity against carcinomic human kidney cells, which are consistent with previous studies, indicating that honey possesses antitumor and anticarcinogenic activities.[59] Different studies have shown the antiproliferative activity of honey on human colon cancer cell line.[60]

The ability to induce tumor cell apoptosis is an important property of a candidate anticancer drug, which discriminates between anticancer drugs and toxic compounds.[61] Our study showed that honey has a significant proliferation inhibitory activity against ACHN cells in a dose-dependent manner [Figure 1]. Much effort has been directed toward the study of the effect of honey on apoptosis and understanding the mechanisms of action. The apoptosis evoked by honey was confirmed by the Annexin-V-FITC [Figure 3]. In the present study, honey-induced apoptosis was involved in cell death. Apoptosis is characterized by distinct morphologic features, including chromatic condensation, cell and nuclear shrinkage, membrane blabbing, and oligonucleosomal DNA fragmentation.[62]

As shown in Figure 3 and 4, honey at higher concentrations (20%) induced significant cell toxicity in the ACHN cells in a dose-dependent manner. Apoptosis only partially contributed in this toxicity, it might be conducted that non-apoptotic cell death to be also involved in honey-induced toxicity in these cells. Although the significance of non-apoptotic cell death in chemotherapy remains largely unclear, it is believed that the non-apoptotic cell death is important under conditions in which apoptosis is inhibited.[63] Mitochondrial and reactive oxygen species--mediated apoptotic mechanisms observed in the antiproliferative activity of honey might have a putative role in the antitumor activity of honey against Ehrlich ascites.[64] The antitumor effect of honey observed in the present investigation was similar to the effect of some other natural products, such as black tea and jacalin, against Ehrlich ascites reported previously. Animals affected with Ehrlich solid carcinoma were treated with a higher concentration of honey (50% v/v) because they were found to be more resistant to treatment with honey as compared with animals affected with ascites. Results demonstrated that even at such a higher concentration of honey, it could not show any drastic change in the growth of Ehrlich solid carcinoma. The antitumor efficacy against solid carcinoma was only 4.34% compared with 39.98% against ascites carcinoma.[65–66] This inefficacy of honey may be due to the systemic effect necessitated by solid carcinoma. To explain further, it is essential to study the exact molecular mechanism behind the antitumor activity of honey. Although the antioxidant property of free radical scavengers of honey has been shown in previous studies,[67,68] carotenoids of high concentrations may act as pro-oxidants in biological systems. Finally, this study showed that honey may contain bioactive compounds that inhibit the proliferation of the renal adenocarcinoma cell line (ACHN) with the involvement of apoptosis or programmed cell death. Further studies are needed to fully recognize the mechanism involved in cell death. Honey could be considered as a promising chemotherapeutic agent in kidney cancer treatment.

## References

[1] Chae EJ, Kim JK, Kim SH, Bae SJ, Cho KS (2005). Renal cell carcinoma: analysis of post-operative recurrence patterns. Radiology.

[2] Motzer RJ, Bander NH, Nanus DM (1996). Renal-cell carcinoma. N Engl J Med.

[3] Vogelzang NJ, Lipton A, Figlin RA (1993). Subcutaneous interleukin-2 plus interferon alfa-2a in metastatic renal cancer: an outpatient multicenter trial. J Clin Oncol.

[4] Minasian LM, Motzer RJ, Gluck L, Mazumdar M, Vlamis V, Krown SE (1993). Interferon alfa-2a in advanced renal cell carcinoma: treatment results and survival in 159 patients with long-term follow-up. J Clin Oncol.

[5] Motzer RJ, Schwartz L, Law TM, Murphy BA, Hoffman AD, Albino AP (1995). Interferon alfa-2a and 13-*cis*-retinoic acid in renal cell carcinoma: antitumor activity in a phase II trial and interaction *in vitro*. J Clin Oncol.

[6] Martel CL, Lara PN (2003). Renal cell carcinoma: current status and future directions. Crit Rev Oncol Hematol.

[7] Sosman JA (2003). Targeting of the VHL-hypoxia-inducible factor hypoxia induced gene pathway for renal cell carcinoma therapy. J Am Soc Nephrol.

[8] Weber KL, Doucet M, Price JE, Baker C, Kim SJ, Fidler IJ (2003). Blockade of epidermal growth factor receptor signaling leads to inhibition of renal cell carcinoma growth in the bone of nude mice. Cancer Res.

[9] Weiss RH, Lin PY (2006). Kidney cancer: identification of novel targets for therapy. Kidney Int.

[10] Wollgast J, Anklam E (2000). Review on polyphenols in Theobroma cacao: changes in composition during the manufacture of chocolate and methodology for identification and Quantification. Food Res Int.

[11] Madhavi DL, Singhai RS, Kulkarni PR, Madhavi DL, Deshpande SS, Salunkhe DK (1996). Food Antioxidants.

[12] Al-Mamary M, Al-Meeri A, Al-Habori M (2002). Antioxidant activities and total phenolics of different types of honey. Nutr Res.

[13] Estevinho L, Pereira A, Moreira L, Dias L, Pereira E (2008). Antioxidant and antimicrobial effects of phenolic compounds extracts of Northeast Portugal honey. Food Chem Toxicol.

[14] Dustmann JH (1979). Antibacterial effect of honey. Apiacta.

[15] Brudzynski K (2006). Effect of hydrogen peroxide on antibacterial activities of Canadian honeys. Can J Microbiol.

[16] Armon PJ (1980). The use of honey in the treatment of infected wounds. Trop.

[17] Effem SE (1988). Clinical observations on the wound healing properties of honey. Br J Surg.

[18] Green AE (1988). Wound healing properties of honey. Br J Surg.

[19] Greenwood D (1993). Honey for superficial wounds and ulcers. Lancet.

[20] Tovey F (2000). Editorial. Honey and sugar as a dressing for wounds and ulcers. Trop Doc.

[21] Molan PC (2001). Potential of honey in the treatment of wounds and burns. Am J Clin Dermatol.

[22] Molan PC (1998). A brief review of honey as a clinical dressing. Primary Intent.

[23] Subrahmanyam M (1991). Topical application of honey in treatment of burns. Br J Surg.

[24] Philips CE (1993). Honey for burns. Gleaning Bee Culture.

[25] Subrahmanyam M, Sahapure AG, Nagane NS, Bhagwat VR, Ganu JV (2001). Effects of topical application of honey on burn wound healing. Ann Burns Fire Disasters.

[26] Keast-Butler J (1980). Honey for necrotic malignant breast ulcers. Lancet.

[27] Mossel DA (1980). Honey for necrotic breast ulcers. Lancet.

[28] Jull A, Walker N, Parag V, Molan P, Rodgers A (2008). Randomized clinical trial of honey-impregnated dressings for venous leg ulcers. Br J Surg.

[29] Molan PC, Betts JA (2008). Using honey to heal diabetic foot ulcers. Adv Skin Wound Care.

[30] Jeddar A, Khassany A, Ramsaroop VG, Bhamjei IE, Moosa A (1985). The antibacterial action of honey: an *in vitro* study. S Afr Med J.

[31] Ali AT, al-Swayeh OA, al-Humayed MS (1997). Natural honey prevents ischemia-reperfusion-induced gastric mucosal lesions and increased vascular permeability in rats. Eur J Gastroenterol Hepatol.

[32] Subrahmanyan M (1993). Storage of skin grafts in honey. Lancet.

[33] Gribel NV, Pashiniski VG (1990). Antitumor properties of honey. Vopr Onkol.

[34] Wattenberg LW, Wattenberg LW, Lipkin M, Boone CW, Kellof GJ (1986). Chemoprevention of cancer by naturally occurring and synthetic compounds. *Cancer Chemoprevention*.

[35] Chinthalapally V, Dhimant D, Barbara S, Nalini K, Shantu A, Bandaru R (1993). Inhibitory effect of caffeic acid esters on azoxymethane-induced biochemical changes and aberrant crypt foci formation in rat colon. Cancer Res.

[36] Swellam T, Miyanaga N, Onozawa M, Hattori K, Kawai K, Shimazui T (2003). Antineoplastic activity of honey in an experimental bladder cancer implantation model: *in vivo* and *in vitro* studies. Int J Urol.

[37] Mosmann T (1983). Rapid colorimetric assay for cellular growth and survival: Application to proliferation and cytotoxicity assays. J Immunol Methods.

[38] Montbriand MJ (2004). Herbs or Natural Products that decrease cancer growth, part one. Oncol Nurs Forum.

[39] Eisenberg DM, Kessler RC, Foster C, Norlock FE, Calkins DR, Delbanco TL (1993). Unconventional medicine in the United States: prevalence, cost, and patterns of use. N Engl J Med.

[40] Montbriand MJ (1994). Decision heuristics of patients with cancer: Alternate and biomedical choices. Unpublished doctoral dissertation. Saskatoon, Saskatchewan.

[41] Montbriand MJ (1995). Alternative therapies as control behaviors used by cancer patients. J Adv Nurs.

[42] Montbriand MJ (1995). Decision tree model des cribbing alternate healthcare choices made by oncology patients. Cancer Nurs.

[43] Montbriand MJ, Heumann LF (1997). Empowerment of seniors through improved communication about medication. Proceedings of the Sixth Science in Health-Social Services for the Elderly and the Disabled.

[44] Montbriand MJ (2000). Alternative therapies: Health professionals’ attitudes. Can Nurs.

[45] Molan PC (1995). The antibacterial properties of honey. Chem in NZ.

[46] Swallow KW, Low NH (1990). Analysis and Quantitation of the carbohydrates in honey using high- performance liquid chromatography. Agric Food Chem.

[47] Sesta G, Persanooddo L, Nisi F, Ricci L (2006). Effects of artificial sugar feeding on sugar composition of royal jelly. Apiacta.

[48] Molan PC, Allen KL (1996). The effect of gamma-irradiation on the antibacterial activity of honey. Pharm Pharmcol.

[49] Galijatovic A, Walle UK, Walle T (2000). Induction of UDP glucuronosyl transferase by the flavonoids chrys in and guercetin in caco- 2 cells. Pharm Res.

[50] Galijatovic A, Otake Y, Walle UK, Walle T (2001). Induction of UDP-glucuronosyltrans ferase UGT1A1 by the flavonoid chrys in Caco-2-Potential role in carcinogen bio inactivation. Pharm Res.

[51] Sabatier S, Amiot MJ, Tacchin M, Aubert S (1992). Identification of flavonoids in sunflower honey. J Food Sci.

[52] Middleton CO, Harbored EJ, Liss AR (1986). Plant flavonoid in biology and medicine- biochemical. pharmacological and structure-activity relationships.

[53] Abuharfeil N, Al-Oran R, Abo-Shehada M (1999). The effect of bee honey on the proliferative activity of human B-and T-Lymphocytes and the activity of phagocytes. Food Agri Immun.

[54] Tonks A, Cooper RA, Price AJ, Molan PC, Jones KP (2001). Stimulation of TNF-alpha release in monocytes by honey. Cytokine.

[55] Rady H (2005). Phytochemical and biological study of an antitumor agent of plant origin mixed withhoney on malignant human cells *in vitro*. Faculity of Science.

[56] Nada O, Ivan B (2004). Honey as cancer-preventive agent. Periodicum Biologorum.

[57] Orsolic N, Terzic S, Sver L, Basic I (2005). Honey-bee products in prevention and/or therapy of murine transplantable tumours. J Sci Food Agri.

[58] Egeblad M, Werb Z (2002). New functions for the matrix metalloproteinases in cancer progression. Nat Rev Cancer.

[59] Abdel Aziz A, Rady H, Amer MA, Kiwan HS (2009). Effect of Some Honey Bee Extracts on the Proliferation, Proteolytic and Gelatinolytic Activities of the Hepatocellular Carcinoma Hepg2 Cell Line. Aus J Basic App Sci.

[60] Jaganathan SK, Mandal M (2009). Honey constituents and its apoptotic effect in colon cancer cells. J Api Product Api Med Sci.

[61] Frankfurt OS, Krishan A (2003). Apoptosis-based drug screening and detection of selective toxicity to cancer cells. Anticancer Drugs.

[62] Kerr JF, Wyllie AH, Currie AR (1972). Apoptosis: A basic biological phenomenon with wide-ranging implications in tissue kinetics. Br J Cancer.

[63] Dinicola S, Cucina A, Pasqualato A, Proietti S, D’Anselmi F, Pasqua G (2010). Apoptosis-inducing factor and caspase-dependent apoptotic pathways triggered by different grape seed extracts on human colon cancer cell line Caco-2. Br J Nutr.

[64] Jaganathan SK, Mandal M (2010). Involvement of non-protein thiols, mitochondrial dysfunction, reactive oxygen species and p53 in honey-induced apoptosis. Invest New Drugs.

[65] Bhattacharyya A, Choudhuri T, Pal S, Chattopadhyay S, K Datta G, Sa G (2003). Apoptogenic effects of black tea on Ehrlich’s ascites carcinoma cell. Carcinogenesis.

[66] Ahmed H, Chatterjee BP, Debnath AK (1998). Interaction and *in vivo* growth inhibition of Ehrlich ascites tumor cells by jacalin. J Biosci.

[67] Beretta G, Orioli M, Facino RM (2007). Antioxidant and radical scavenging activity of honey in endothelial cell cultures. Planta Med.

[68] Vilma B, Petras RV, Violeta Č (2007). Radical scavenging activity of different floral origin honey and beebread phenolic extracts. Food Chem.

